# Potential of subdermal solar energy harvesting for medical device applications based on worldwide meteorological data

**DOI:** 10.1117/1.JBO.26.3.038002

**Published:** 2021-03-10

**Authors:** Maximilien V. Tholl, Adrian Zurbuchen, Hildegard Tanner, Andreas Haeberlin

**Affiliations:** a University of Bern, sitem Center for Translational Medicine and Biomedical Entrepreneurship, Bern, Switzerland; b Bern University Hospital, Department of Cardiology, Bern, Switzerland

**Keywords:** medical implant, energy harvesting, subdermal solar cell, modeling, open source code

## Abstract

**Significance:** Active implants require batteries as power supply. Their lifetime is limited and may require a second surgical intervention for replacement. Intracorporal energy harvesting techniques generate power within the body and supply the implant. Solar cells below the skin can be used to harvest energy from light.

**Aim:** To investigate the potential of subdermal solar energy harvesting.

**Approach:** We evaluated global radiation data for defined time slots and calculated the output power of a subdermal solar module based on skin and solar cell characteristics. We assumed solar exposure profiles based on daily habits for an implanted solar cell. The output power was calculated for skin types VI and I/II.

**Results:** We show that the yearly mean power in most locations on Earth is sufficient to power modern cardiac pacemakers if 10 min midday solar irradiation is assumed. All skin types are suitable for solar harvesting. Moreover, we provide a software tool to predict patient-specific output power.

**Conclusions:** Subdermal solar energy harvesting is a viable alternative to primary batteries. The comparison to a human case study showed a good agreement of the results. The developed code is available open source to enable researchers to investigate further applications of subdermal solar harvesting.

## 1 Introduction

Active electronic implants are increasingly used for therapeutic and diagnostic purposes. Therapeutic devices include, e.g., cardiac pacemakers, cochlear,^1^ or retinal^2^ implants. Examples for diagnostic devices are implantable cardiac loop recorders^3^ or continuous glucose-monitoring systems.^4^ Most devices rely on an internal battery with a limited lifetime. The most common active electronic implant^5^ is the cardiac pacemaker with more than one million implantations per year.^6^ The power requirement of a modern single chamber pacemaker is below 10  μW.^7^ The average battery lifetime of VVI-programmed pacemakers was identified as 7.2 years^8^ while the median survival time of patients which required permanent cardiac pacing was found to be 8.5 years.^9^ This mismatch contributes to the high replacement rate of 25% for cardiac pacemakers.^6^

A possible solution for the problem of depleting batteries is presented by subdermal solar cells, enabling to recharge implants using solar or artificial light. The main aging cause for solar cells is ultraviolet irradiance,^10^ which is blocked by the skin in subdermal applications. Therefore, the expectable durability of the solar modules is high. The potential of subdermal solar cells as a power source for electronic implants has been studied extensively with Monte Carlo simulations of light distribution through human skin (mainly skin type VI)^11^ using in-house code,^12^^–^^14^ in ex – and in vivo animal trials. In a human case study,^15^ a wearable device with a solar module below optical filters that mimick the light transmission through human skin of type I/II was used to record the achievable output power of a subdermal solar module. An ex vivo trial covered a solar module with a porcine skin flap and analyzed the generated power.^16^ The in vivo animal trials were performed in mice^17^^,^^18^ and pigs.^19^^,^^20^ All trials reported promising results in the range of 1.9  to $10\;\mathrm{mW}\;{\mathrm{cm}}^{-2}$ of generated power by subdermal solar modules at an implantable size scale under midday solar irradiation (AM1.5G). Furthermore, researchers are aiming at using the photodiodes of retinal implants with ambient or artificial light to supply the implant with power.^21^ However, the influence of seasons, weather, and location on Earth on the achievable output power of subdermal solar cells has not been investigated yet.

In this study, we are evaluating the potential of subdermal solar cells in combination with solar radiation. We use worldwide meteorological data in combination with human skin and solar cell characteristics to calculate the power output of a subdermal photovoltaic module over a whole year. Our methodology and the developed python code is available in a GitHub repository: github.com/matholl/SolarHarvestMeteoEval under GNU General Public License v3.0. This will provide others with a tool to not only reproduce our results but also to model the theoretical output power from subdermal solar energy harvesting for different skin types, solar cell characteristics, solar exposure profiles, and years of interest.

## 2 Methods

**Fig. 1:**
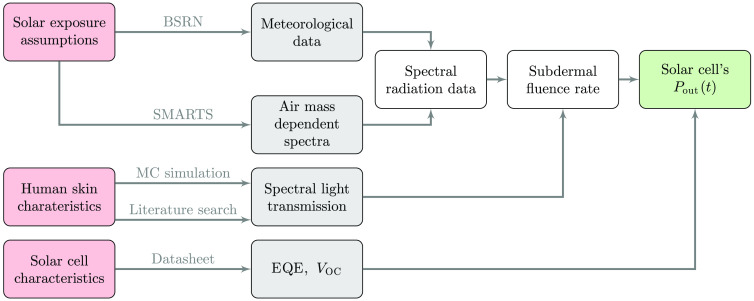
Flowchart of the meteorological data evaluation. The method requires three inputs (red boxes), which determine the data (gray boxes) for the subsequent calculations. The intermediate results are indicated by the white boxes and lead to the final result, the solar cell’s output power (green box). The following abbreviations were used: BSRN, baseline surface radiation network; SMARTS, simple model of the atmospheric radiative transfer of sunshine; MC, Monte Carlo; EQE, external quantum efficiency; $V_{\mathrm{OC}}$, open circuit voltage; $P_{\text{out}}(t)$, time-dependent power output.

We developed a model that uses meteorological data to estimate the output power of a subdermal solar module. The model requires three different inputs (solar exposure assumptions, human skin, and solar cell characteristics), which are indicated in red in Fig. 1. The global radiation data are provided by the baseline surface radiation network (BSRN).^22^ The measurements take place at ground level (elevation of the measurement station), with a temporal resolution of 1 to 3 min per datapoint. Since the BSRN does not provide spectral data, the simple model of the atmospheric radiative transfer of sunshine (SMARTS)^23^ is used to model the spectral composition of the global horizontal irradiance based on input parameters such as location, time and air mass (AM). The spectral composition of the irradiance is needed because the skin’s light transmission and the solar cell’s efficiency are dependent on the wavelength. Combined, the meteorological data and AM dependent irradiance spectra generate time and location-dependent spectral irradiation data that can be used for subsequent calculations. The spectral light transmission of human skin can be characterized using Monte Carlo (MC) simulations of the light propagation in skin models^11^ or by direct measurements as described in the literature.^24^ This allows calculating the subdermal fluence rate dependent on the irradiance. The fluence rate is defined as “at a given point in space, the radiant power incident on a small sphere, divided by the cross-sectional area of that sphere.”^25^ The resulting subdermal fluence rate was used to calculate the theoretical output power of a subdermal solar module based on the given solar cell characteristics [external quantum efficiency (EQE) and open circuit voltage ($V_{\mathrm{OC}}$)]. The subsequent sections present the methodology introduced in Fig. 1 in more detail.

### 2.1 Solar Exposure Assumptions

**Fig. 2:**
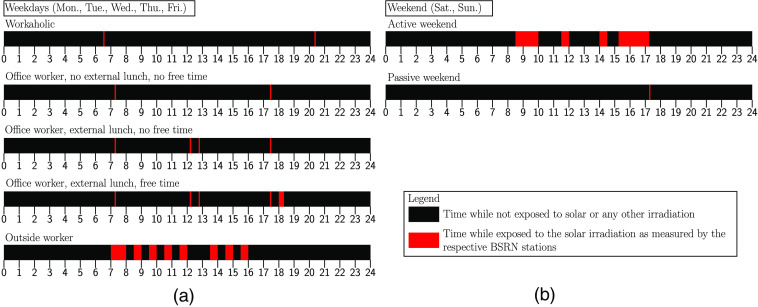
Solar irradiation profiles based on arbitrary lifestyle assumptions. The timeslots for which each profile are exposed to solar irradiation is marked in red along a 24-h timeframe for (a) weekdays and (b) weekends. The narrowest strip of red equates to 5 min of solar exposure.

Solar irradiation exposure profiles for humans with different daily routines have to be defined to estimate the achievable energy output of subdermally implanted solar modules. We defined arbitrary timeslot-profiles for different daily routines as shown in Fig. 2. The daily routines were separated into five different profiles during weekdays accounting for different professions and habits. The solar exposure times per day range from 10  min/day at very early and late hours during the day for workaholic office workers up to 4.5  h/day for outside workers. Furthermore, we assumed two different weekend solar irradiation exposure profiles with 4.5  h/day representing an active day outside and 5  min/day for a day mainly indoors. A table with the exact solar exposure times for the different profiles is provided in Appendix A.

### 2.2 Human Skin Characteristics

**Fig. 3:**
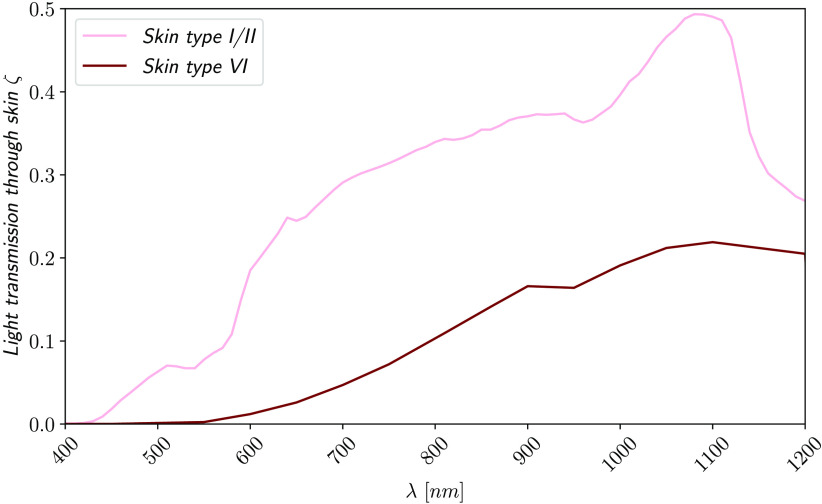
Comparison of the light transmission through 2.5 mm of skin type I/II^24^ and type VI.^11^

The light transmission of human skin depends on its thickness and composition. In an extensive *in silico* study,^11^ we calculated the fraction of light absorbed by a solar module in different depths within the skin. We assumed a 2.5 mm thick,^15^^,^^24^ skin type VI with high light absorption and scattering^11^ to calculate the worst case scenario in terms of light transmission. Further information on the Monte Carlos simulations is given in Appendix C. We included skin type I/II^24^ in our calculations for comparison. Figure 3 shows a comparison of the light transmission through the two evaluated skin types. All realistic skin types should be between the two given transmission spectra, except for albinos which will have higher light transmissions.

### 2.3 Solar Cell Characteristics

**Fig. 4:**
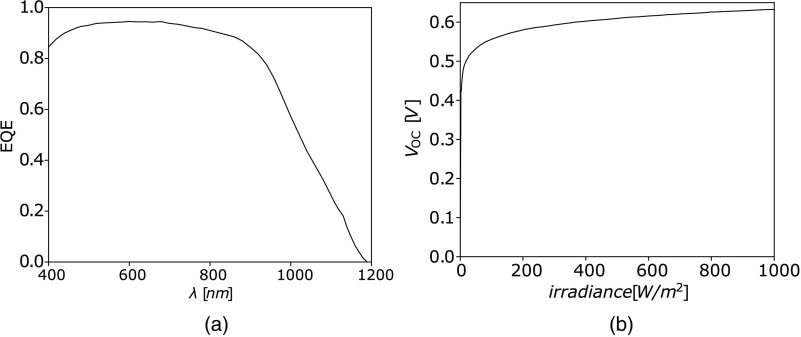
(a) EQE and (b) $V_{\mathrm{OC}}$ of a standard monocrystalline silicon solar cell.^26^

The EQE is unique to each solar cell type and is defined as the wavelength-dependent ratio of the number of collected electrons at the cell’s contacts to the number of incident photons on the solar cell. Moreover, the cell’s open circuit voltage ($V_{\mathrm{OC}}$) is dependent on the irradiance. The output power of a cell can be calculated if both EQE and the $V_{\mathrm{OC}}$ are known. Figure 4 shows the EQE and $V_{\mathrm{OC}}$ of a standard monocrystalline silicon solar cell.^26^

### 2.4 Spectral Radiation Modelling

The calculation of light transmission through human skin and the subdermal solar module’s output power requires spectral information. However, the BSRN database only provides global radiation data in the form of a general power per area value but no spectral information. Therefore, the spectral distribution of BSRN’s global radiation needs to be modeled.

The AM describes the relative path length of light through Earth’s atmosphere and is defined as given in Eq. (1). The shortest possible way is reached when the sun is at zenith, which corresponds to an AM equal to 1:

$$\mathrm{AM}=\frac{1}{\cos(\theta )}.$$ (1)

**Fig. 5:**
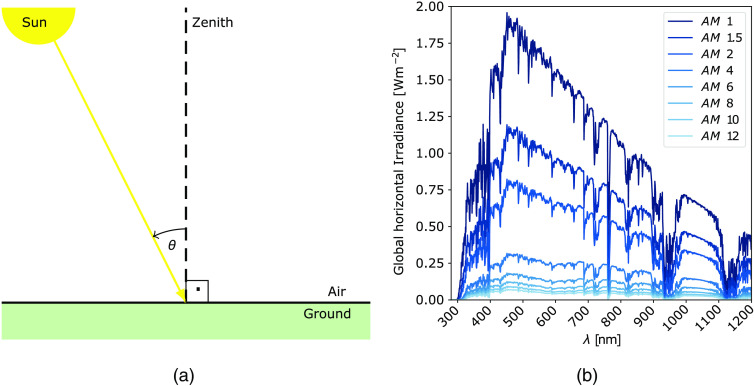
The influence of (a) the sun’s zenith angle θ and (b) the resulting air mass on Earth’s ground level spectral irradiance modeled by the simple model of the atmospheric radiative transfer of sunshine.

The zenith angle is defined as the angle of the sun’s elevation with respect to a vertical position above the observer on Earth (Fig. 5). The zenith angle can be calculated knowing the position on Earth (longitude, latitude) and time (exact time within the yearly cycle). The full formula for the zenith angle is derived from the National Oceanic and Atmospheric Administration’s booklet *General Solar Position Calculations*.^27^ Increasing zenith angles correspond to longer paths for sunlight through the atmosphere and therefore to significant attenuation in intensities (Fig. 5). To analyze the influence of different pathlengths through Earth’s atmosphere on the resulting spectral irradiance, we use the SMARTS model. It predicts clear-sky spectral irradiances^28^ and allows analyzing the impact of various atmospheric parameters on the spectral irradiance on Earth. The SMARTS results for varying AMs (as shown in Fig. 5) are used to calculate the spectral irradiation from the BSRN station’s global radiation values.

### 2.5 Calculation of the Solar Cell’s Output Power

The output power ($P_{\text{out}}$) of a solar module is calculated according to Eq. (2). We assumed an active area ($A_{\mathrm{SC}}$) of $3.6\;{\mathrm{cm}}^{2}$ for our calculations to be able to compare it to a human case study.^15^ Moreover, we assumed the EQE and $V_{\mathrm{OC}}$ of a standard moncrystalline solar cell as indicated in its datasheet.^26^ The outer dimensions of such a solar module would be 22×21×1.8  mm. The solar cell’s fill factor (FF) is a property that is corresponding to the maximum output power. FF is typically around 0.8 for highly efficient monocrystalline silicon cells.^29^ The solar cell’s $V_{\mathrm{OC}}$ depends on the subdermal fluence rate, which can be interpreted as the implanted cell’s irradiance:

$$P_{\text{out}}=\mathrm{FF}\;V_{\mathrm{OC}}I_{\mathrm{SC}}A_{\mathrm{SC}}.$$ (2)

The short circuit current ($I_{\mathrm{SC}}$) of the solar cell at a certain implantation depth (scd) is calculated as described in Eq. (3) (q is the elementary charge):

$$I_{\mathrm{SC}}(\lambda ,\mathrm{scd})=\int_{\lambda =400\;\mathrm{nm}}^{1200\;\mathrm{nm}}\phi (\lambda )\zeta (\lambda ,\mathrm{scd})\mathrm{EQE}(\lambda )qd\lambda .$$ (3)

The spectral irradiance on the patient’s skin is calculated from the meteorological data and the SMARTS modeling results. The fluence rate under the skin, which is harvested by the solar cell is calculated by multiplying the spectral irradiance with the light transmission through skin (ζ) in a depth of 2.5 mm in skin type VI^11^ or type I/II^24^ for comparison. The photon flux (ϕ) can be derived from the subdermal fluence rate and the photon energy at the respective wavelength.

### 2.6 Comparison to a Human Case Study

We chose to evaluate the mean output power for all BSRN stations in the year 2015 to compare our results to the outcome of a human case study by Bereuter et al.^15^ The study was conducted in different locations within Switzerland and in different seasons of the same year. Volunteers were asked to wear a data logger with a $3.6\text{-}{\mathrm{cm}}^{2}$ solar module of monocrystalline silicon cells covered with optical filters that mimic the light transmission of skin type I/II with a thickness of 2.5 mm on their arm. The data logger recorded the output power of the “subdermal” solar cells during their daily life and enables the comparison of our results and a human case study.

## 3 Results

**Fig. 6:**
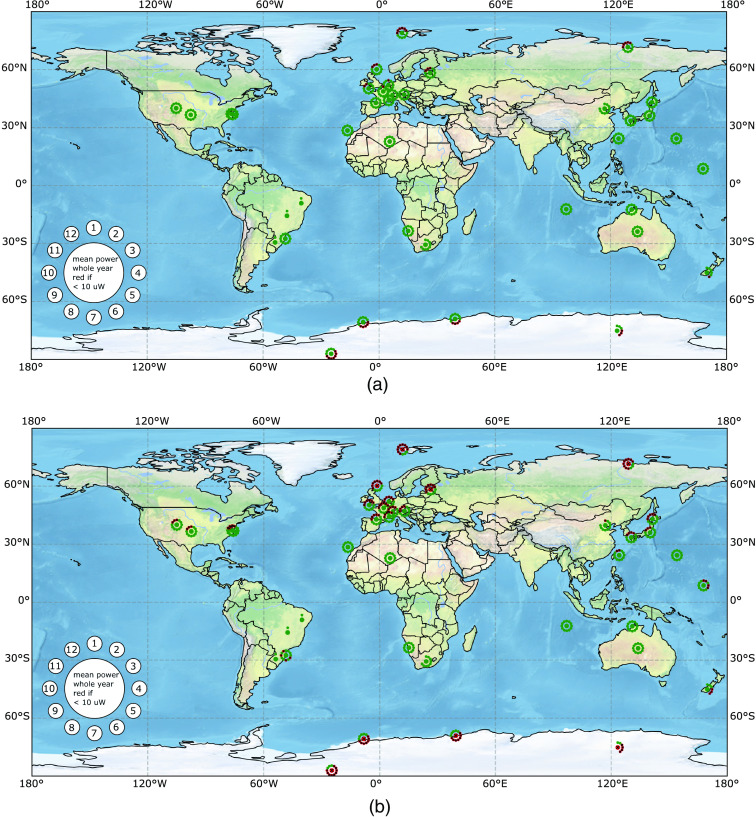
Resulting output power of a $3.6\;{\mathrm{cm}}^{2}$ subdermal solar module in 2.5 mm depth for skin types (a) I/II and (b) VI when exposed to the solar irradiation profile “office worker, external lunch, no free time” during weekdays, with no exposure during weekends (see Fig. 2). The mean output power of each month and the whole year is displayed with a flower-like symbol as indicated in the lower left of the map. The central circle indicates the yearly mean power and the small circles indicate the monthly mean power according to the numbers in the legend where “1” corresponds to January and “12” to December. The yearly and monthly circles are green if the mean power surpasses 10  μW, the power requirement of a modern cardiac pacemaker,^7^ and red with a black cross if they do not. Missing month symbols are due to incomplete datasets of the BSRN network.

Our model resulted in time-dependent output power for the exposure profiles given in Fig. 2. These output powers are available for all weather stations that contributed measurements to the BSRN database. We calculated the mean output power for all available months and the mean power of the whole year for all stations. The exposure-specific output powers are visualized on a world map with a binary color code (Fig. 6). The station-specific results of all exposure profiles were saved in tables and plotted in separate figures. The digital appendix provides detailed data for each individual weather station in the form of tables and plots, world maps for each solar exposure profile as in Fig. 6, the full python code and documentation.

### 3.1 Mean Output Power Worldwide for One Exemplary Exposure Profile

The resulting mean output power for all evaluated BSRN stations is shown in Fig. 6. The results of every station are plotted in a flower-shaped label. The big central circle is green or red with a black cross if the mean output power of the subdermal solar module is above or below the necessary power of 10  μW to power modern cardiac single chamber pacemakers, respectively. Similarly, the 12 small circles represent the mean output power from January (1) to December (12). Small circles without color indicate a lack of data. The flower-like, color-coded symbols are plotted on a world map on the position of the station. The calculations assumed a subdermal monocrystalline silicon solar module with an active area of $3.6\;{\mathrm{cm}}^{2}$ in 2.5 mm implantation depth. The evaluated time slots of the “office worker, external lunch, no free time” exposure profile during weekdays and no exposure during weekends (compare Fig. 2) was chosen. Figure 6 compares the resulting output power of such a subdermal solar module for skin type I/II and type VI. The skin type I/II results are shown in Fig. 6. The yearly mean output power (central circle) of the subdermal solar module under skin type I/II surpasses the power threshold for modern pacemakers for all meteorological stations. The winter months close to the poles with insufficient output power have to be bridged with an appropriate energy storage. The yearly mean output power of the subdermal solar module under skin type VI (c.f. Fig. 6) is still sufficient for the latitudes of approximately 55°N until 60°S. Compared to the skin type I/II results, the number of months that need to be bridged by an energy storage are increased.

### 3.2 Mean Output Power for All User Profiles in Central Europe

To exemplify the influence of the solar exposure profiles, we show the mean output power of the different exposure profiles in Payerne, Switzerland, in 2015 for a $3.6\text{-}{\mathrm{cm}}^{2}$ solar module under 2.5-mm skin type I/II in Table 1. The results show that the yearly mean power of the subdermal solar module is sufficient for all exposure profiles except for the extreme cases with very short irradiation in the early mornings or late evenings. The minimum mean power requirement of 10  μW for modern cardiac pacemakers was surpassed by a factor of almost 5 as soon as 10 min of midday solar irradiation were assumed during weekdays (office worker with external lunch and no free time). The output power of a light skinned outside worker’s subdermal solar module surpasses the minimum power requirement by a factor of 60. The variability of the available mean power is considerable for the different seasons of the year, which has to be considered while optimizing the solar module area and the capacity of the energy buffer storage (e.g., a rechargeable battery or a supercapacitor).

**Table 1:** Mean power in μW of the $3.6\;{\mathrm{cm}}^{2}$ standard monocrystalline silicon subdermal solar module exposed to solar irradiation under 2.5 mm of skin type I/II according to the described profiles in Payerne, Switzerland, in 2015.

Months	Workaholic mean P (μW)	Office worker + int. lunch mean P (μW)	Office worker + ext. lunch mean P (μW)	Office worker + ext. lunch + free time mean P (μW)	Outside worker mean P (μW)	Active weekend mean P (μW)	Passive weekend mean P (μW)
1	0.00	0.00	14.88	14.88	166.74	26.60	0.00
2	0.00	0.71	24.97	24.98	303.14	39.05	0.29
3	0.00	5.14	48.84	52.16	587.22	88.99	0.44
4	1.33	14.86	72.37	86.35	909.95	84.99	1.95
5	4.18	14.10	59.28	76.07	780.82	206.06	3.21
6	6.66	22.18	86.12	112.70	1079.64	190.13	1.71
7	5.70	24.97	93.96	127.01	1162.01	182.58	2.42
8	1.65	15.28	68.01	86.17	828.28	174.11	1.57
9	0.40	8.05	55.55	60.41	677.72	114.07	1.20
10	0.00	1.07	28.98	28.99	358.89	56.85	0.12
11	0.00	0.00	20.10	20.10	232.92	40.76	0.00
12	0.00	0.00	13.34	13.34	140.73	27.10	0.00
Full year	1.66	8.86	48.87	58.60	602.34	102.61	1.08

### 3.3 Open Source Python Code

We supply the full developed python code under a GNU General Public License v3.0. The necessary software to run the code is available open source. A python version 3 installation with several common libraries (matplotlib, numpy, datetime, csv, cartopy, …) is necessary to run the code. The BSRN database is accessible in Ref. 30 and can be downloaded via File Transfer Protocol (ftp). The path to the local folder containing the BSRN data needs to be given in the first lines of the “main.py” file. Moreover, the first lines of the “main.py” file allows to adapt the year of evaluation, the solar cell area, type, and skin type. Furthermore, the solar exposure assumptions can be changed in a table that is loaded by the code (c.f. Appendix A).

## 4 Discussion

The developed python code allows evaluating any subdermal solar module and skin combination for many locations worldwide and in all years that are available in the BSRN database (1992 to today). Moreover, it allows to evaluate and optimize the subdermal solar energy harvesting solution (solar module type, active area, and implantation depth) patient-specific based on the individual’s skin characteristics and solar exposure habits. We were able to show that our method is able to produce realistic results that are very close to a human case study, as demonstrated in Sec. 4.1.

### 4.1 Comparison to a Human Case Study

Our simulation results can be compared to the real-life measurements acquired by Bereuter et al.^15^ in the same year as described in Sec. 2. Table 2 summarizes the seasonal and yearly mean power of our study and the human study.

**Table 2:** Comparison of our calculated and the measured^15^ mean power in μW of the $3.6\;{\mathrm{cm}}^{2}$ standard monocrystalline silicon subdermal solar module exposed to solar irradiation under 2.5 mm of skin type I/II in Payerne, Switzerland, in 2015. The seasonal means were calculated as defined in the human case study.

Time frame	Office worker + ext. lunch + weekend mean power (μW)	Office worker + ext. lunch + free time + weekend mean power (μW)	Bereuter et al.^15^ mean power (μW)
Summer	105.41	131.34	106
Autumn	52.72	55.14	66
Winter	20.64	20.64	27
Yearly	61.66	71.39	67

The recorded mean power over all seasons and subjects in the human study was 67  μW. Our predicted yearly mean output powers of the profiles “office worker + ext. lunch” (48.87  μW) and “office worker + ext. lunch + free time” (58.60  μW) are close to the observed overall mean output power in the human case study (67  μW). Additional 12.73  μW would be generated assuming that the two mentioned exposure profiles would spend one weekend day per month according to the “active weekend” profile and the rest of the weekend days according to the “passive weekend” profile. This would result in 61.66 and 71.39  μW respectively for the above mentioned exposure profiles, which closely resembles the results in the human case study.

The monthly mean power measured in the human study was 106  μW during summer, 66  μW during autumn, and 27  μW during winter. Our predicted mean output power values for the two office worker profiles with external lunch in all seasons are similar to the reported mean power in the human study (see Table 2). The predicted output power slightly overestimated the mean power in summer and underestimated it during autumn and winter compared to the results in the human case study. This might be due to a shift of the exposed time slots in real life. In summer, direct solar exposure at mid-day may be too hot. However, in autumn and winter, direct solar exposure is usually rarer, less intense, and therefore more pleasant than in summer. This might lead to increased mid-day exposures.

The measured mean peak mean power for one day was 744  μW in the human case study. We predict mean output powers of up to 1.16 mW for outside workers in summer. This difference might be due to a lower solar exposure time of the volunteer or due to our assumption that the implant is always facing the sun during the exposed time slots.

Our predicted output power of the subdermal solar module could be adjusted by changing the arbitrary solar exposure profiles. Regardless, we were able to show that the predicted output power for reasonable solar exposure profiles were very close to the observations of Bereuter et al. in their human case study which used the same solar cells, active area, and skin transmission in the same country and year.

### 4.2 Solar Module Optimization

We assumed a monocrystalline silicon solar cell with an active area of $3.6\;{\mathrm{cm}}^{2}$ for our calculations. This allows a direct comparison to the human study of Bereuter et al.^15^ However, the solar module is not yet optimized for subdermal applications and can be improved as suggested in the following sections.

#### 4.2.1 Solar cell type

**Fig. 7:**
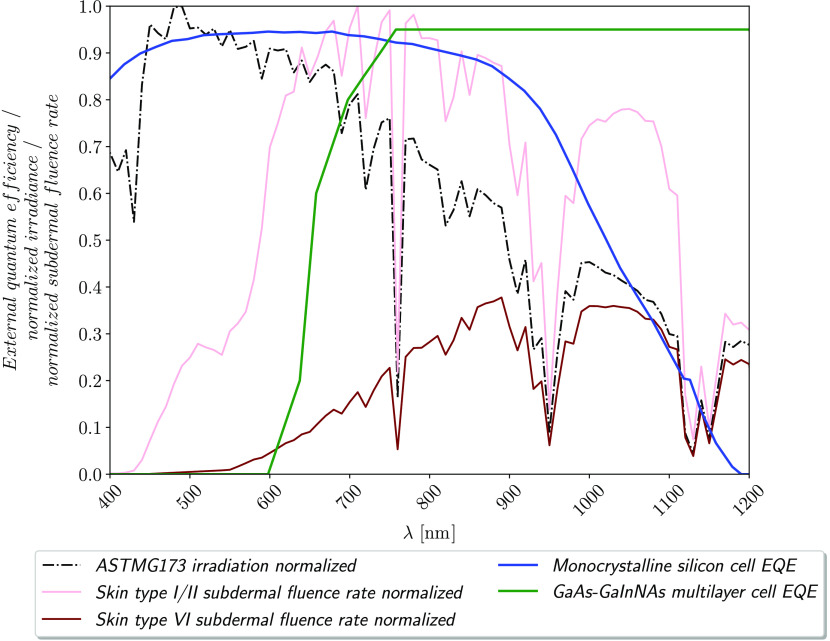
Comparison of the normalized standard irradiation ASTMG173 for midday solar irradiation in mid-latitudes (black, dash-dotted), the resulting subdermal fluence rate for skin type I/II and type VI (pink and brown), and the external quantum efficiencies of monocrystalline solar cells and the adapted GaAs-GaInNAs multilayer (blue and green). The subdermal fluence rates were normalized to the maximum value of the two fluence rates.

The available light below the skin differs from normal solar irradiation. The output power of subdermal solar cells may be increased using solar cells with a different spectral sensitivity than the one of standard monocrystalline silicon solar cells. This can be achieved using other semiconductor materials or using quantum dots.^31^ An adapted double junction GaAs-GaInNAs solar cell would show high efficiency in the wavelength range of 650 to 1200 nm,^32^ which is more suitable for subdermal applications. Figure 7 shows a comparison of the normalized standard irradiation ASTMG173, the resulting subdermal fluence rate for light and skin type VI and the external quantum efficiencies of monocrystalline solar cells and the adapted GaAs-GaInNAs multilayer cell. ASTMG173 is the terrestrial reference spectrum ASTMG173^33^ for photovoltaic performance evaluation in mid latitudes (30°-60° N or S^34^). It assumes an air mass of 1.5 (θ=48  deg) and irradiance on a sun-facing tilted (37 deg) surface. The global irradiance on that surface is $1000\;{Wm}^{-2}$. The GaAs-GaInNAs solar cell’s EQE shows more overlap with the subdermal fluence rate for skin type VI compared to a conventional monocrystalline silicon solar cell which would lead to an increased output power of the subdermal solar module by 50% (see Appendix B.1, B.2). However, its sensitivity from 550 to 650 nm is not optimal for the light skin’s fluence rate. This could be improved using quantum dots. Our results show that even without quantum dots, the GaAs-GaInNAs cell would be more efficient than a standard silicon solar cell for skin type VI and type I/II (see Appendixes 7.3 and 7.4).

#### 4.2.2 Solar cell area minimization

The solar cell area is directly proportional to the output power [see Eq. (2)]. The solar cell size should be minimized for aesthetic reasons and patient comfort. The minimum power requirement of the electronic implant can be used as a threshold for the active area calculation of the solar module.

### 4.3 Active Charging

Subdermal solar cells can actively recharge electronic implants using an artificial light source, similar to inductive charging. A recently published study^18^ investigated the concept of active photonic power transfer and presented its feasibility during an *in vivo* trial. The study suggested the use of a skin attachable light emitting patch that constantly illuminates the subdermal solar cell at a wavelengths 670 nm, because the energy per photon and the skin transmittance presented in the study are both high. The optimal wavelength for photonic power transfer changes for different skin types and solar cell technologies (compare Fig. 7). The wavelength range of 600 to 800 nm would be reasonable for photonic power transfer through skin type I/II using monocrystalline silicon solar cells. Both investigated solar cells can supply the baseline energy via solar charging and enable active charging if necessary.

### 4.4 Limitations

The sunlight exposure profiles of Fig. 2 are selected arbitrarily, because human behavior patterns are not generalizable. The arbitrary selected exposures shall illustrate extreme examples with very low or high solar exposure, as well as mixed scenarios. Optimal implantation sites within the body for subdermal solar cells are hairless, sun-exposed sites as, e.g., the neck, arms, and hands.

The aforementioned SMARTS model (see Sec. 2.4), which is used to model the spectral irradiance depending on varying air masses, predicts clear sky irradiances only. However, the BSRN stations measure data independent of the cloud coverage.

Most of the BSRN stations record the global radiation using a pyranometer, which is mounted horizontally with a field of view of 180 deg. Therefore, the measurement device is always facing the sun. Consequently, we assume that the implant is always facing the sun during the exposed time slots shown in Fig. 2. However, this simplifying assumption does not account for shadows or situations in which the skin above the implant is not facing the sun. The assumed exposure times are purposefully chosen very short to account for that simplification.

In this study, we only consider exposure to sunlight. We do not account for exposure to artificial light in buildings and therefore underestimate the total exposure to light. Even though artificial light is usually significantly less powerful than sunlight, the energy generated by illumination of indoor lighting may not negligible.^15^

The generated energy of the subdermal solar cells should be stored in an energy buffer as for example a rechargeable battery. The charging and discharging process of the battery should be optimized for the regular charging and discharging cycles.

### 4.5 Future Work

This section presents a possible solution for the aforementioned limitations which could be implemented in future work. The open-source software approach will allow experts in every field to review and contribute to the improvement of the model.

The sunlight exposure profiles were selected arbitrarily to represent a wide range of different lifestyles. There are considerable differences across different cultures and among individuals and a universal, worldwide lifestyle description is not possible. A quantitative description of the solar exposure of typical pacemaker patients in a society with similar solar exposure could be attempted by measuring light exposure^35^ or mobility via GPS-tracking.^36^

The modeling of the solar irradiation’s spectral distribution is based on the clear sky assumption of SMARTS. Simulating the influence of cloud coverage on the spectral distribution of the solar irradiation would increase the accuracy.

Our model currently does not model shadows or other irradiation extenuating situations (e.g., wearing of clothes above the implant). A possibility to model these events would be to introduce a virtual neutral density filter with a situation specific attenuation factor above the implant. A recent study proposed an analytical approach to model partial shading conditions of photovoltaic arrays.^37^ The orientation of the implant toward the sun can be modeled using the distinction of direct and diffuse light measured by BSRN stations.

Furthermore, indoor illumination exposure profiles could be added to the model. The spectral radiation from indoor light sources can differ significantly. A distinction between typical home and office lighting might be necessary.

Modeling of the energy buffer system including the power management module would simulate a system closer to a fully working implant.

## 5 Conclusion

Our results show that subdermal solar energy harvesting bears the potential to power modern electronic implants for all skin types and in most locations on Earth. Our method and the developed open source python code allows to evaluate other subdermal solar cells, skin types, or solar exposure assumptions for all years since 1992 and many locations worldwide. Moreover, it allows to evaluate patient-specific settings as skin type and layer thicknesses and specific solar exposure assumptions as in Fig. 2. We were able to show that our assumptions lead to a result that is very close to a human case study. An optimization of the solar cell’s spectral sensitivity for subdermal applications improves the output power, especially for darker skin types.

## 6 Appendix A: User Profiles

**Fig. 8:**
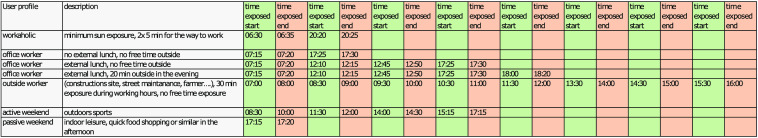
Description of the different solar exposure time slot assumptions that were used in the meteorological data evaluation. Each exposure profile contains pairs of daytimes which define a solar exposure slot.

Figure 8 describes the time slots at which different user profiles were assumed to be exposed to solar irradiation. The developed model fetches meteorological irradiation data for the adjustable time slots and evaluates the generated power output of a subcutaneous solar module.

## 7 Appendix B: Potential of Solar Cell Optimization

### 7.1 Comparison of Solar Cell Resulting Output Power on World Map for Skin Type VI

**Fig. 9:**
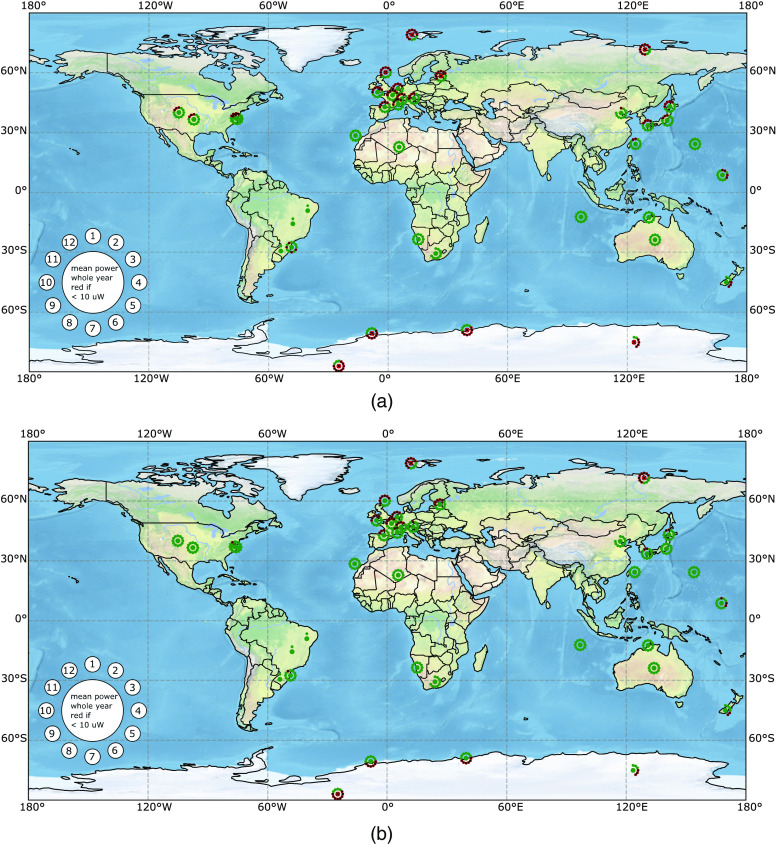
Resulting output power of different $3.6\text{-}{\mathrm{cm}}^{2}$ subdermal solar modules (a) monocrystalline silicon solar module, (b) multilayer GaAs-GaInNAs solar module in 2.5 mm depth of skin type VI when exposed to the solar irradiation profile “office worker, external lunch, no free time” during weekdays, with no exposure during weekends (see Fig. 2). The mean output power of each month and the whole year is displayed in the same way as in Fig. 6.

The comparison of the resulting yearly mean output power of two different solar cells under skin type VI is shown in Fig. 9. Figure 9(a) shows the results of a standard monocrystalline solar cell and Fig. 9(b) shows the results for a GaAs-GaInNAs multilayer solar cell. The yearly mean output power of the subdermal, standard monocrystalline silicon solar module’s output power is above 10  μW for all meteorological stations within latitudes of 55°N to 60°S. The subdermal GaAs-GaInNAs multilayer solar module’s yearly mean output power is above 10  μW for all meteorological stations within latitudes of 60°N to 70°S. Compared to the results of the monocrystalline silicon solar cell, the number of months that needs to be bridged by an energy storage decreased. Moreover, only stations close to poles are not able to generate a yearly sufficient mean power. The mean output power of the GaAs-GaInNAs multilayer solar cell is higher than the one of the monocrystalline silicon cell as visible near Earth’s poles.

### 7.2 Comparison of Solar Cell Resulting Output Power in Table for Skin Type VI

Tables 3 and 4 show the resulting output power for the different exposure profiles in Payerne, Switzerland, in 2015, for a subdermal solar cell under skin type VI.

**Table 3:** Mean power in μW of the $3.6\;{\mathrm{cm}}^{2}$ subdermal standard monocrystalline silicon solar module exposed to solar irradiation under 2.5 mm of skin type VI according to the described profiles in Payerne, Switzerland, in 2015.

Months	Workaholic mean P (μW)	Office worker + int. lunch mean P (μW)	Office worker + ext. lunch mean P (μW)	Office worker + ext. lunch + FT mean P (μW)	Outside worker mean P (μW)	Active weekend mean P (μW)	Passive weekend mean P (μW)
1	0	0	3.88	3.88	42.7	14.7	0
2	0	0.079	6.43	6.43	78.7	23	0.114
3	0	1.23	12.7	13.2	153.03	43.2	0.271
4	0.276	3.9	18.9	22.4	238.14	64.8	0.831
5	1.06	3.63	15.5	19.8	203.47	114.52	1.55
6	1.76	5.8	22.6	29.5	281.91	90.8	1.19
7	1.5	6.52	24.5	33.2	303.41	91.4	1.36
8	0.411	4.01	17.7	22.5	216.40	79.1	0.944
9	0.003	2.07	14.5	15.4	177.12	61.3	0.45
10	0	0.177	7.47	7.47	92.9	31.2	0.02
11	0	0	5.23	5.23	60.4	23.5	0
12	0	0	3.45	3.45	35.3	12.4	0
Full year	0.42	2.28	12.7	15.2	156.95	54.2	0.56

**Table 4:** Mean power in μW of the $3.6\;{\mathrm{cm}}^{2}$ multilayer subdermal GaAs-GaInNAs solar module exposed to solar irradiation under 2.5 mm of skin type VI according to the described profiles in Payerne, Switzerland, in 2015. The power ratio in the last row is defined as the ratio of yearly mean output powers of the multilayer GaAs-GaInNAs solar cell to the monocrystalline silicon solar cell.

Months	Workaholic mean P (μW)	Office worker + int. lunch mean P (μW)	Office worker + ext. lunch mean P (μW)	Office worker + ext. lunch + FT mean P (μW)	Outside worker mean P (μW)	Active weekend mean P (μW)	Passive weekend mean P (μW)
1	0	0	5.78	5.78	63.5	21.9	0
2	0	0.116	9.59	9.59	117.373	34.3	0.17
3	0	1.82	18.9	19.7	228.375	64.4	0.404
4	0.411	5.81	28.2	33.5	355.786	96.8	1.24
5	1.58	5.41	23.2	29.6	304.538	171.26	2.32
6	2.62	8.64	33.8	44.1	422.171	135.826	1.78
7	2.23	9.73	36.8	49.7	454.288	136.708	2.03
8	0.613	5.98	26.6	33.7	323.541	118.189	1.41
9	0.00398	3.09	21.7	23	264.457	91.5	0.675
10	0	0.262	11.2	11.2	138.5	46.5	0.029
11	0	0	7.8	7.8	90	35	0
12	0	0	5.15	5.15	52.6	18.5	0
Full year	0.621	3.4	19	22.7	234.597	80.9	0.838
Power ratio	1.49	1.49	1.50	1.49	1.49	1.49	1.49

### 7.3 Comparison of Solar Cell Resulting Output Power on World Map for Skin Type I/II

**Fig. 10:**
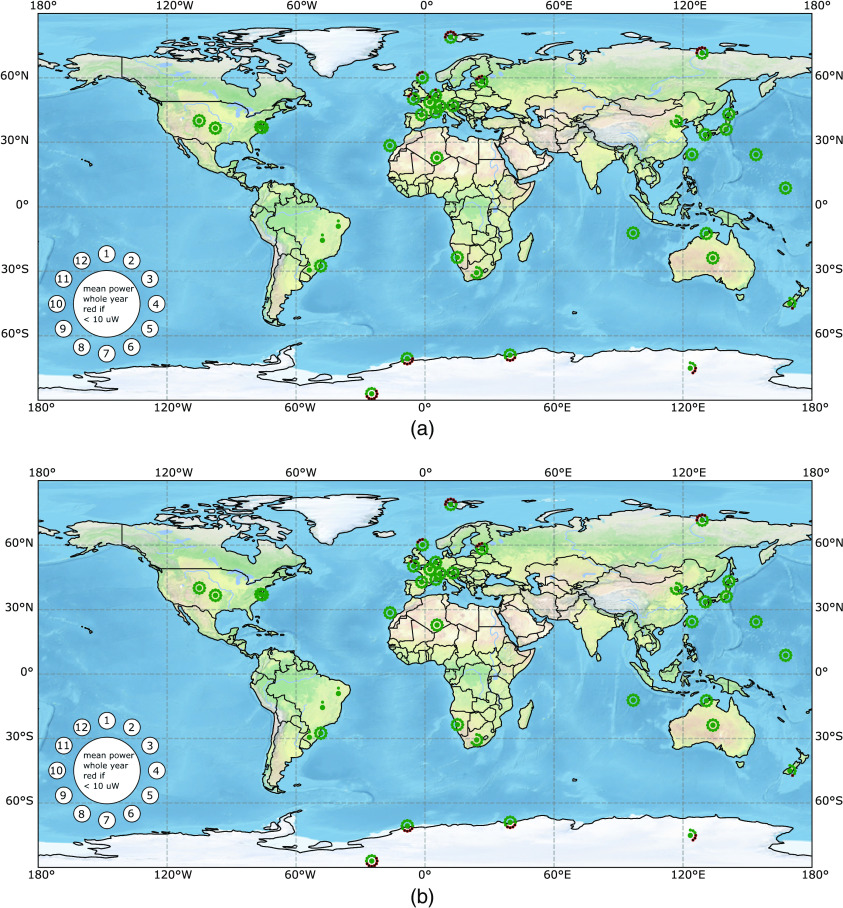
Resulting output power of different $3.6\text{-}{\mathrm{cm}}^{2}$ subdermal solar modules (a) monocrystalline silicon solar module and (b) multilayer GaAs-GaInNAs solar module in 2.5 mm depth of skin type I/II when exposed to the solar irradiation profile “office worker, external lunch, no free time” during weekdays, with no exposure during weekends (see Fig. 2). The mean output power of each month and the whole year is displayed in the same way as in Fig. 6.

The comparison of the resulting yearly mean output power of two different solar cells under skin type I/II is shown in Fig. 10. Figure 10(a) shows the results of a standard monocrystalline solar cell and Fig. 10(b) shows the results for a GaAs-GaInNAs multilayer solar cell. The mean output power of the GaAs-GaInNAs multilayer solar cell is higher than the one of the monocrystalline silicon cell, but this is not visible on our binary color code. Refer to the digital appendix or Appendix 7.4 for absolute output power numbers.

### 7.4 Comparison of Solar Cell Resulting Output Power in Table for Skin Type I/II

Tables 5 and 6 show the resulting output power for the different exposure profiles in Payerne, Switzerland, in 2015, for a subdermal solar cell under skin type I/II.

**Table 5:** Mean power in μW of the $3.6\;{\mathrm{cm}}^{2}$ subdermal standard monocrystalline silicon solar module exposed to solar irradiation under 2.5 mm of skin type I/II according to the described profiles in Payerne, Switzerland, in 2015.

Months	Workaholic mean P (μW)	Office worker + int. lunch mean P (μW)	Office worker + ext. lunch mean P (μW)	Office worker + ext. lunch + FT mean P (μW)	Outside worker mean P (μW)	Active weekend mean P (μW)	Passive weekend mean P (μW)
1	0	0	14.9	14.9	166.74	26.6	0
2	0	0.707	25	25	303.141	39.1	0.291
3	0	5.14	48.8	52.2	587.217	89	0.437
4	1.33	14.9	72.4	86.3	909.95	85	1.95
5	4.18	14.1	59.3	76.1	780.825	206.064	3.21
6	6.66	22.2	86.1	112.698	1079.638	190.129	1.71
7	5.7	25	94	127.012	1162.01	182.575	2.42
8	1.65	15.3	68	86.2	828.279	174.113	1.57
9	0.405	8.05	55.6	60.4	677.716	114.075	1.2
10	0	1.07	29	29	358.892	56.8	0.12
11	0	0	20.1	20.1	232.915	40.8	0
12	0	0	13.3	13.3	140.73	27.1	0
Full year	1.66	8.86	48.9	58.6	602.338	102.608	1.08

**Table 6:** Mean power in μW of the $3.6\;{\mathrm{cm}}^{2}$ multilayer subdermal GaAs-GaInNAs solar module exposed to solar irradiation under 2.5 mm of skin type I/II according to the described profiles in Payerne, Switzerland, in 2015. The power ratio in the last row is defined as the ratio of yearly mean output powers of the multilayer GaAs-GaInNAs solar cell to the monocrystalline silicon solar cell.

Months	Workaholic mean P (μW)	Office worker + int. lunch mean P (μW)	Office worker + ext. lunch mean P (μW)	Office worker + ext. lunch + FT mean P (μW)	Outside worker mean P (μW)	Active weekend mean P (μW)	Passive weekend mean P (μW)
1	0	0	17.6	17.6	199.518	71.1	0
2	0	0.852	29.5	29.5	359.343	107.522	0.734
3	0	6.22	57.6	61.6	693.218	196	1.36
4	1.62	17.8	85.4	102.304	1072.353	292.119	3.8
5	5.03	16.6	69.7	89.9	918.13	515.002	7.01
6	8.02	26.2	101.245	132.759	1268.943	408.676	5.43
7	6.86	29.5	110.457	149.715	1366.078	411.154	6.16
8	2	18.2	80.1	101.995	975.099	356.757	4.27
9	0.487	9.72	65.6	71.5	799.395	276.692	2.11
10	0	1.3	34.2	34.2	424.695	143.438	0.212
11	0	0	23.7	23.7	277.533	109.451	0
12	0	0	15.7	15.7	169.239	59.9	0
Full year	2	10.5	57.6	69.2	710.295	245.653	2.59
Power ratio	1.20	1.19	1.18	1.18	1.18	2.39	2.40

## 8 Appendix C: Information on Previous Monte Carlo Simulations

**Fig. 11:**
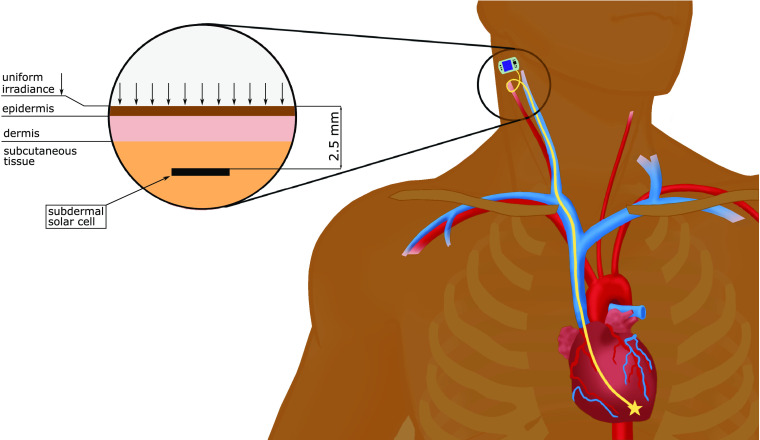
Illustration of an implanted pacemaker powered by subcutaneous solar cells. The detail view shows the model geometry of the Monte Carlo simulations in a previous publication.^11^ The MC simulations modelled the light propagation in humas skin tissue (skin type VI) for uniform irradiance at wavelengths 400 to 1200 nm.

This section of the appendix provides additional information on the previously published Monte Carlo simulations of skin type VI.^11^ Figure 11 shows the imagined concept of a subdermal solar pacemaker. The detail view shows a two-dimensional cross section of the model geometry with a uniform irradiance on human skin of type VI and its layers with an implanted solar cell.

The Monte Carlo simulations were performed using an in-house developed code.^12^^–^^14^ Important input parameters for the simulations are the following variables: absorption coefficient $\mu_{a}$, reduced scattering coefficient $\mu_{s^{\prime }}$, anisotropy g, and index of refraction n for all layers.

Table 7 shows the volume fractions (VFs) of the skin’s constituents in the different layers. The epidermis is a bloodless layer containing melanosomes, which are producing melanin and are therefore responsible for the skin color and type. Melanin is highly light absorbent and therefore important for the light transmission. The dermis layer contains blood vessels and is considerably thicker than the epidermis. The subcutaneous tissue layer contains mainly fat cells and is vascularized.

**Table 7:** VF of the skin’s constituents within the three layers used as input for the published Monte Carlo simulations.

Tissue	Blood VF	Water VF	Fat VF	Melanosome VF	Other VF
Epidermis (type VI)	0^38^	0.65^38^	0.1^38^	0.0165^39^	0.2335^40^
Dermis	0.02	0.75	0.2	0.01	0.02
Subcutaneous tissue	0.08	0.7	0.2	0.0095	0.105

The VFs and their respective $\mu_{a}$ are used to calculate an average layer specific $\mu_{a}$ according to Eq. (4) which is then used in the MC simulation:

$$\mu_{a}=\sum VF_{i}\mu_{a,i}.$$ (4)

The reduced scattering coefficient can be determined based on constants a and b in the empirical Eq. (5):^41^

$$\mu_{s^{\prime }}=a{\left(\frac{\lambda }{500}\right)}^{-b}.$$ (5)

The coefficients a and b are given in literature and summarized in Table 8.

**Table 8:** Coefficients a and b of Eq. (5), describing the skin layers’ $\mu_{s^{\prime }}$.^41^

Tissue	a	b
Epidermis (VI)	80	1.15
Dermis	60	1.4
Subcutaneous tissue	40	1

The anisotropy factor g was set to 0.9 for all tissues. Measurements of the refractive indices resulted in 1.34 for the epidermis layer and 1.41 for dermis and subcutaneous tissue layer.^42^

Please refer to Ref. 43 to get more detailed information on the Monte Carlo simulations.

## Data Availability

All data will be available in the digital appendix. Any potentially missing data will be available upon request. The procedure and calculations are available in the open source python code on GitHub (github.com/matholl/SolarHarvestMeteoEval).

## Glossary

BSRN Baseline Surface Radiation Network

SMARTS Simple Model of the Atmospheric Radiative Transfer of Sunshine

AM Air Mass

MC Monte Carlo

θ Zenith angle [rad]

ASTMG173 Terrestrial reference spectrum for photovoltaic performance evaluation in mid latitudes

λ Wavelength [*nm*]

ζ Light transmission through skin

ϕ Photon flux [#$photons\;s^{-1}\;m^{-2}$]

EQE External Quantum Efficiency

$V_{OC}$ Open circuit voltage [V]

$I_{SC}$ Short circuit current [A]

scd Solar cell implantation depth [*mm*]

q Elementary charge [C]

$P_{\text{out}}$ Output power [W]

$A_{SC}$ Active area of a solar cell [$m^{2}$]

FF Fill factor
